# Identification of bronchoalveolar and blood immune-inflammatory biomarker signature associated with poor 28-day outcome in critically ill COVID-19 patients

**DOI:** 10.1038/s41598-022-13179-0

**Published:** 2022-06-09

**Authors:** Guillaume Voiriot, Karim Dorgham, Guillaume Bachelot, Anne Fajac, Laurence Morand-Joubert, Christophe Parizot, Grigorios Gerotziafas, Dominique Farabos, Germain Trugnan, Thibaut Eguether, Clarisse Blayau, Michel Djibré, Alexandre Elabbadi, Aude Gibelin, Vincent Labbé, Antoine Parrot, Matthieu Turpin, Jacques Cadranel, Guy Gorochov, Muriel Fartoukh, Antonin Lamazière

**Affiliations:** 1 Assistance Publique—Hôpitaux de Paris, Service de médecine intensive réanimation, Hôpital Tenon, Sorbonne Université, 4 rue de la Chine, 75020 Paris, France; 2 Département d’immunologie, INSERM Centre d’Immunologie Et Des Maladies Infectieuses CIMI-Paris, Assistance Publique—Hôpitaux de Paris, Hôpital Pitié-Salpêtrière, Sorbonne Université, Paris, France; 3 Département de biochimie, INSERM Centre de Recherche Saint-Antoine (CRSA), Assistance Publique—Hôpitaux de Paris, Hôpital Saint-Antoine, Sorbonne Université, Paris, France; 4 Assistance Publique—Hôpitaux de Paris, Service d’anatomie et cytologie pathologiques, Hôpital Tenon, Sorbonne Université, Paris, France; 5 INSERM Institut Pierre Louis d’épidémiologie et de Santé Publique, Assistance Publique—Hôpitaux de Paris, Laboratoire de VirologieHôpital Saint-Antoine, Sorbonne Université, Paris, France; 6 INSERM U938 “Cancer, Haemostasis and Angiogenesis” Centre de Recherche Saint-Antoine, Assistance Publique—Hôpitaux de Paris, Service d’hématologie biologique, Hôpital Tenon, Sorbonne Université, Paris, France; 7 Assistance Publique—Hôpitaux de Paris, Service de pneumologie, Hôpital Tenon, Sorbonne Université, Paris, France

**Keywords:** Antimicrobial responses, Cytokines, Inflammation, Innate immunity, Lipidomics, Lipids, Metabolomics, Clinical microbiology, SARS-CoV-2

## Abstract

The local immune-inflammatory response elicited by severe acute respiratory syndrome coronavirus 2 (SARS-CoV-2) infection is still poorly described, as well as the extent to which its characteristics may be associated with the outcome of critical Coronavirus disease 2019 (COVID-19). In this prospective monocenter study, all consecutive COVID-19 critically ill patients admitted from February to December 2020 and explored by fiberoptic bronchoscopy with bronchoalveolar lavage (BAL) were included. Biological assays, including digital ELISA cytokine profiling and targeted eicosanoid metabolomic analysis, were performed on paired blood and BAL fluid (BALF). Clinical outcome was assessed through the World Health Organization 10-point Clinical Progression Scale (WHO-CPS) at the 28th day (D28) following the admission to intensive care unit. A D28-WHO-CPS value higher than 5 defined a poor outcome. Seventy-six patients were included, 45 (59%) had a poor day-28 outcome. As compared to their counterparts, patients with D28-WHO-CPS > 5 exhibited a neutrophil-predominant bronchoalveolar phenotype, with a higher BALF neutrophil/lymphocyte ratio, a blunted local type I interferon response, a decompartimentalized immune-inflammatory response illustrated by lower BALF/blood ratio of concentrations of IL-6 (1.68 [0.30–4.41] vs. 9.53 [2.56–19.1]; p = 0.001), IL-10, IL-5, IL-22 and IFN-γ, and a biological profile of vascular endothelial injury illustrated by a higher blood concentration of VEGF and higher blood and/or BALF concentrations of several vasoactive eicosanoids. In critically ill COVID-19 patients, we identified bronchoalveolar and blood immune-inflammatory biomarker signature associated with poor 28-day outcome.

## Introduction

During the coronavirus disease 2019 (COVID-19) pandemic caused by severe acute respiratory syndrome coronavirus 2 (SARS-CoV-2), it has been observed that less than 3% of individuals who are infected with the virus require hospital care^1^. Among them, up to one third develop the severe form of the disease, mainly acute respiratory failure, requiring admission to an intensive care unit (ICU)^2–4^ with an in-ICU mortality ranging from 28 to 42% in Europe^5–8^. In this severely affected population, an altered immuno-inflammatory systemic response has been described, with a marked systemic release of pro-inflammatory cytokines and an impaired interferon (IFN) type-1 response^9–12^, but with important differences at the individual level^13^. Given these findings, therapeutic targets have been proposed and immunomodulatory drugs have been investigated for SARS-CoV-2 infection. However, despite intensive research efforts, corticosteroids and tocilizumab remain the only medication that suggest a mortality benefit in randomized controlled trials^14^. This highlights the need to deepen our pathobiological understanding of the host immune-inflammatory response elicited by SARS-CoV-2 infection. Specifically, a better characterization of the immune-inflammatory response within affected lungs is warranted. To date, knowledge from studies in patients with severe COVID-19 describes perturbations of all cellular subpopulations in the lung microenvironment^15,16^ and high concentrations of pro-inflammatory cytokines within the epithelial lining fluid^17,18^, with a high heterogeneity among patients. Investigations in clinical settings are needed to better characterize the bronchoalveolar cellular landscape and the biochemical characteristics of the local host response and to establish the extent to which this local signature may be associated with the course of severe COVID-19.

To gain insight into this issue, we report observations of bronchoalveolar lavage (BAL) in 76 COVID-19 patients admitted to the ICU of a University teaching hospital in Paris, France during the first two waves of the pandemic in 2020. We focused on characterizing the cellular and biochemical patterns of the local host response. We hypothesized that some bronchoalveolar and blood immune-inflammatory biomarkers might be associated with a poor 28-day outcome in critical COVID-19.

## Methods

### Study design and patient selection

We conducted a comprehensive observational monocenter study in the ICU of Tenon Hospital in Paris, France. From February 15th to December 15th, 2020, all adult patients with PCR-confirmed SARS-CoV-2 infection on nasopharyngeal swabs or lower respiratory tract specimens were screened, and those having undergone a fiberoptic bronchoscopy with BAL were included.

### Data collection

Demographics, comorbidities, clinical and routine laboratory parameters, radiological findings, and microbiological investigations were collected on ICU admission, as well as outcomes and therapeutic management, including medical therapies and organ supports during ICU stay.

Severity at admission was assessed through the Simplified Acute Physiology Score (SAPS) II^19,20^, the Sequential Organ Failure Assessment (SOFA) score^21^ and the World Health Organization 11-point Clinical Progression Scale (WHO-CPS)^22^.

Immunocompromised status was defined by at least one of the following conditions: splenectomy, HIV infection, long-term steroid therapy, other long-term immunosuppressive therapy, solid organ transplantation, and malignant hemopathy or cancer.

Clinical outcome was assessed through the WHO-CPS on the 28th day following ICU admission (D28-WHO-CPS).

### Sample collection

Fiberoptic bronchoscopy with BAL was performed by clinician’s decision as part of routine care. The procedure consisted of instillation/suction of three consecutive syringe volumes (50 mL each) of sterile saline into a distal bronchus during fiberoptic bronchoscopy. The washed territory was usually the most abnormal on chest imaging. The first syringe (5 to 20 mL) of re-aspired bronchoalveolar lavage fluid (BALF) was dedicated exclusively to bacteriological investigations (for more details regarding the microbiological workup, see the electronic supplementary material (ESM)). A BALF sample (minimum 15 mL) was systematically sent to the cytology laboratory, with a paired blood sample (EDTA) for concomitant cytological analysis. The remaining collected BALF was dispensed in dry tubes for routine laboratories (virology, mycology, parasitology, see the ESM).

Routine blood tests were performed daily as part of routine care. On the day of BAL, blood from dry tubes was centrifuged at 1600 rpm for 7 min at 4 °C, and serum was aliquoted (200 µL) and stored at − 80 °C less than 2 h after sampling for biochemical investigations.

### Cytological analysis (BALF and blood)

The total cell count of BALF was determined using a KOVA cell chamber. BALF was then centrifuged (200 g for 5 min at 10 °C), the cell pellet was diluted in phosphate buffered saline, and May–Grunwald–Giemsa-stained cytocentrifuge preparations (Cytospin 3; Shandon Scientific, Cheshire, UK) were performed to assess differential cell counts (macrophages, lymphocytes, and neutrophils). BALF cell-free supernatants were stored at − 80 °C less than 2 h after sampling.

Blood and BALF (same-day samples) lymphocyte phenotyping were performed as part of routine care. In fresh whole blood, the absolute count and percentage of lymphocyte subpopulations were measured with an AQUIOS flow cytometer (Beckman Coulter) as per manufacturer instructions for use. BALF lymphocyte phenotyping was done after filtration, two washes of BALF cells, and staining with TetraCHROME CD45-FITC/CD4-PE/CD8-ECD/CD3-PC5 antibody cocktail (Beckman Coulter, #6607013). Data were acquired and analyzed on a Navios flow cytometer (Beckman Coulter).

### Biochemical investigations (BALF and blood)

#### Cytokines

Cytokine concentrations were measured in frozen/thawed BALF cell-free supernatant and serum (same-day samples). Cytokine abbreviations are all gathered in the ESM. The Simoa™ (single molecule array) HD-1 analyzer (Quanterix, Lexington, MA, USA) was used for the ultrasensitive immunodetection (digital ELISA) of granulocyte–macrophage colony-stimulating factor (GM-CSF), interleukin-2 (IL-2), interferon-α (IFN-α), and vascular endothelial growth factor (VEGF) using single-plex bead-based assays, according to manufacturer instructions. The Quanterix® SP-X™ imaging and analysis platform allowed determination of the concentrations of IL-1β, IL-4, IL-5, IL-6, IL-8, IL-10, IL-12p70, IL-22, IFN-γ, and tumor necrosis factor-α (TNF-α) using the Human CorPlex Cytokine Panel Array from Quanterix Corporation. B-cell activating factor (BAFF) concentrations were quantified using ELISA kits from R&D Systems (Bio-Techne, Lille, France). Each cytokine concentration in the samples was interpolated from the calibration curve by multiplying it by the dilution factor, and the concentration was then expressed in pg/mL. Samples with non-detectable values were evaluated according to the limit of detection value (LOD); samples above the detection range were replaced by the upper limit of quantification (ULOQ).

#### Eicosanoids

Eicosanoid metabolomic analysis was adapted and performed, as previously described elsewhere, in serum^23,24^ and in BALF cell-free supernatants^25^. Eicosanoid abbreviations are all gathered in the ESM. Briefly, 100 µL of serum or 1 ml of BALF cell-free supernatant was supplemented with an internal standard mix (Cayman Chemical, Interchim France) consisting of 6 deuterated species (for details, see the ESM). Lipid metabolites were isolated by solid phase extraction from a 60 mg Strata X column (Phenomenex, Le Pecq, France). The extracted samples were evaporated and reconstituted in 100 µL of mobile phase (H20/ACN/Acetic Acid, 60/40/0.02). The eicosanoids were separated by reversed-phase liquid chromatography using a Kintex Evo C18 column (Phenomenex). The molecular species were analyzed by tandem quadrupole mass spectrometer (6500QTrap, ABSciex, Les Ulis France) operated in the negative-ionization mode via multiple-reaction monitoring (113 MRM) of M-H > Fragment transitions that were optimized for selectivity and sensitivity^26^. Quantification was performed using MultiQuant 2.1 software (ABSciex). Twenty-five external standards (for details, see the ESM) were assayed under the same conditions, and quantitation was achieved by the stable isotope dilution method. External standards included two eicosanoid mixtures prepared by Cayman Chemical (for details, see the ESM). Semi-quantitative analyses were performed for the other lipid species, including 82 molecular species.

### Data processing and statistical analysis

Patients were grouped according to their D28-WHO-CPS. A value higher than 5 (D28-WHO-CPS > 5) defined a poor 28-day outcome, whereas a value equal to or lower than 5 (D28-WHO-CPS ≤ 5) defined a good 28-day outcome. A value higher than 5 meant either that the patient needed organ support(s) (high flow oxygen or non-invasive ventilation or mechanical ventilation, and/or dialysis, and/or vasopressors, and/or extra-corporeal membrane oxygenation) or that the patient was deceased.

Continuous data were expressed as median [first through third quartiles] and were compared using the non-parametric Mann–Whitney test. Categorical data were expressed as a number (percentage) and were evaluated using the chi-square test or Fischer exact test. Two-sided p values less than 0.05 were considered significant.

Biological data were combined and subjected to multivariate analysis and regression versus the 28-day WHO-CPS category. The raw data were processed with multivariate data analysis SIMCA 15 software (Sartorius-Umetrics, Västerbotten, Sweden). Prior to modeling, a principal component analysis (PCA) was achieved in order to define the major trends in the overall variability of the biological data using a non-supervised approach. Secondly, orthogonal partial least-squares discriminant analysis (OPLS-DA) was performed after scaling the variables with unit-variance (UV) scaling. Such a statistical analysis was conducted to assess whether immune-inflammatory biomarkers from the BALF and blood are associated with the 28-day outcome.

### Ethical considerations

This study was approved by the institutional review board of Sorbonne University (Reference CER-2020-54) according to French regulations. All participants (or their relatives) gave consent to participate.

## Results

### Patient selection

**Figure 1:**
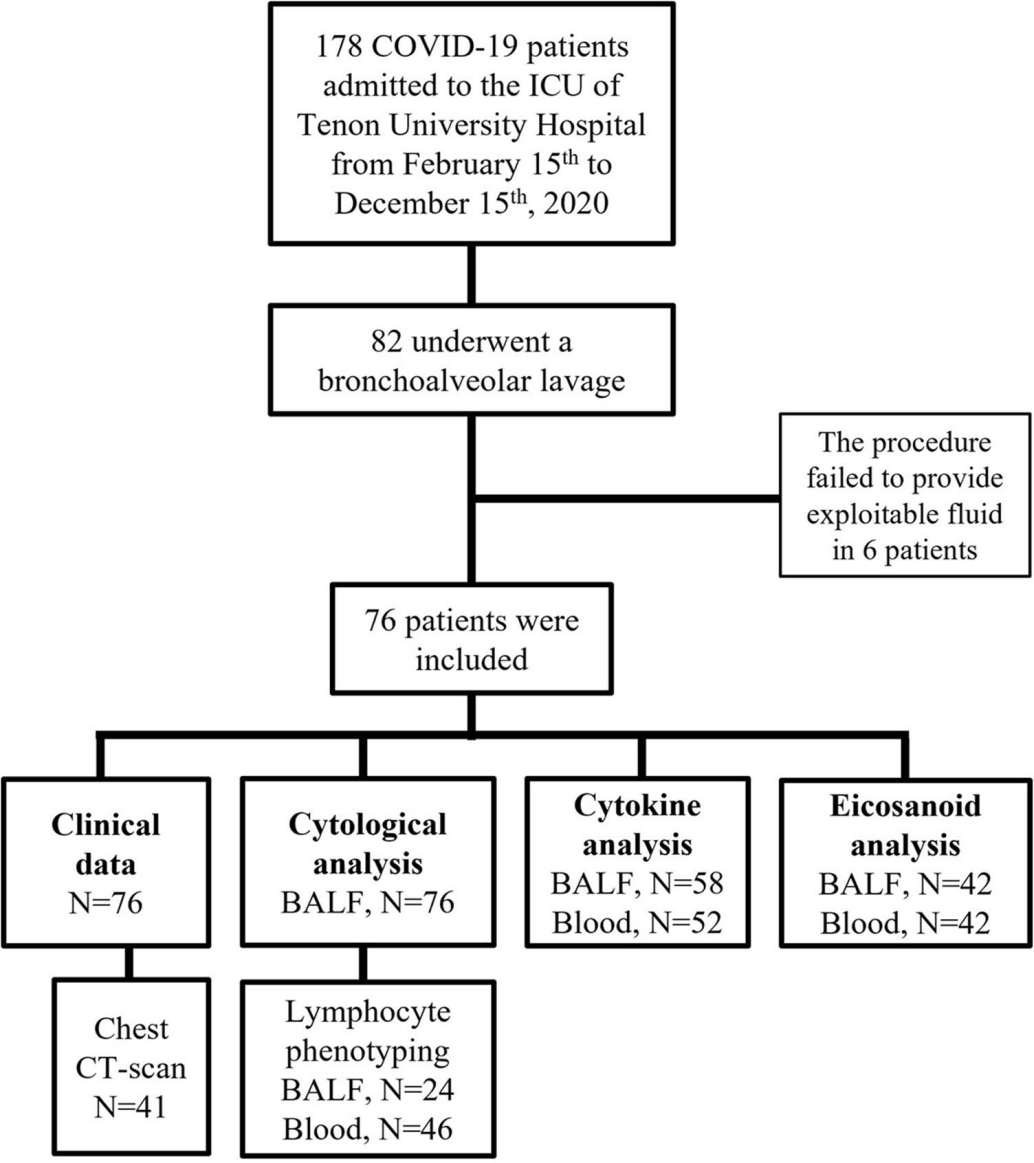
Flowchart. *BALF* bronchoalveolar lavage fluid, *Chest CT-scan* chest computed tomography scan, *ICU* intensive care unit.

During the study period, 178 consecutive patients with severe COVID-19 were admitted to the ICU of Tenon Hospital, 82 of whom underwent a fiberoptic bronchoscopy with BAL, at a median time of 2.5 [1-6] days after ICU referral and 13 [9-17] days after symptom onset. The main reason for performing a fiberoptic bronchoscopy with BAL was to rule out a coinfection and/or a superinfection, especially when respiratory tract secretions were scarce, which made it difficult for clinicians to collect endotracheal aspirate. During the first wave of the pandemic in France (February 15th to May 31st), 39/102 (38%) patients underwent a fiberoptic bronchoscopy with BAL, as compared with 43/76 (57%) during the second wave (June 1st to December 15th). The procedure failed to provide exploitable fluid in 6 patients. The remaining 76 patients (38 recruited during each wave) were studied (Fig. 1).

### Clinical characteristics and outcomes of the study population

Baseline characteristics of the study population, management, and outcomes are reported in Table 1. Briefly, the patients included 54 (71%) males, were aged 64 [52–70] years, were moderately overweight, and frequently had comorbidities and immunosuppression (n = 21; 28%). Forty-five (59%) patients had a D28-WHO-CPS > 5. As compared with their counterparts, patients with a poor 28-day outcome were older and less likely to be obese.

**Table 1:** Baseline characteristics, management and outcomes of critically ill COVID-19 patients, according to the 28-day World Health Organization 10-point Clinical Progression Scale (WHO-CPS).

Patients	All patients (n = 76)	D28 WHO-CPS > 5 group (n = 45)	D28 WHO-CPS ≤ 5 group (n = 31)	*P *value^a^
Age (year)	64 [52–70]	66 [62–73]	58 [49–68]	**0.002**
Sex male	54 (71)	34 (76)	20 (65)	0.32
Diabetes	24 (32)	18 (40)	6 (19)	0.08
Arterial hypertension	44 (58)	27 (60)	17 (55)	0.81
ARB or ACEi	33 (43)	19 (42)	14 (45)	0.82
Body Mass Index > 30 (kg/m^2^)	28 (37)	12 (27)	16 (52)	**0.03**
Immunocompromised status^b^	21 (28)	13 (29)	8 (26)	0.8
Time from symptoms onset to hospital admission (days)	6 [4–9]	6 [4–9]	6 [4–9]	0.61
Time from symptoms onset to ICU admission (days)	9 [6–11]	10 [6–12]	8 [5–10]	0.21
**Severity on ICU admission**				
SAPS II	36 [25–47]	37 [27–51]	34 [25–43]	0.33
SOFA score	7 [5–10]	7 [5–11]	7 [6–10]	0.84
WHO–CPS	6 [6–8]	6 [6–8]	6 [6–8]	0.68
PaO_2_/FiO_2_ (mmHg)^c^	120 [93–175]	118 [88–173]	127 [100–186]	0.53
Vasopressors	30 (39)	20 (44)	10 (32)	0.34
Maximum dose of norepinephrine^d^	0.22 [0.16–0.34]	0.25 [0.20–0.38]	0.14 [0.10–0.27]	**0.04**
Acute kidney injury^e^	27 (36)	21 (47)	6 (19)	**0.02**
**Blood measurements on ICU admission**				
C-reactive protein (mg/L)^f^	173 [101–272]	177 [105–275]	163 [60–266]	0.27
Procalcitonin (ng/L)^f^	0.64 [0.22–1.70]	0.91 [0.31–3.27]	0.45 [0.21–1.49]	0.07
Lymphocytes (G/L)	0.73 [0.49–1.03]	0.62 [0.48–0.94]	0.76 [0.53–1.07]	0.33
**Organ supports during ICU stay**				
Invasive mechanical ventilation	72 (95)	45 (100)	27 (87)	**0.03**
Prone position	59 (78)	41 (91)	18 (58)	**0.001**
ECMO	3 (4)	1 (2)	2 (6)	0.56
Vasopressors	51 (67)	37 (82)	14 (45)	**0.001**
Renal replacement therapy	15 (20)	12 (27)	3 (10)	0.08
**Specific therapies during ICU stay**				
Corticosteroid	65 (86)	39 (95)	26 (84)	0.75
Tocilizumab	9 (12)	5 (11)	4 (13)	1
**Complications during ICU stay**				
Pulmonary embolism	10 (13)	6 (13)	4 (13)	1
ICU-acquired pneumonia	51 (67)	37 (82)	14 (45)	**0.001**
Other ICU-acquired infection	6 (8)	6 (13)	0 (0)	0.08

Data are presented as median [first through third quartiles] or number (%). Patients were grouped according to their WHO-CPS value at 28-day. A score value higher than 5 (D28-WHO-CPS > 5) defined a poor 28-day outcome, whereas a score value equal or lower than 5 (D28-WHO-CPS ≤ 5) defined a good 28-day outcome.

*ARB* angiotensin receptor blocker, *ACEi* angiotensin-converting enzyme inhibitor, *ECMO* extra-corporeal membrane oxygenation, *ICU* intensive care unit, *SAPS II* simplified acute physiologic score II, *PaO2/FiO2* ratio of the partial pressure of oxygen/inspired fraction of oxygen, *SOFA* sequential organ failure assessment, *WHO-CPS* World Health Organization 10-point clinical progression scale.

^***a***^P values refer to differences between D28-WHO-CPS > 5 and D28-WHO-CPS ≤ 5 groups. Boldface values indicate statistically significant differences between the groups.

^***b***^Immunocompromised patients were distributed as followed: long-term corticosteroid, n = 8; kidney transplant, n = 7; cancer, n = 3; HIV, n = 2; sickle cell, n = 1.

^***c***^Patients received either invasive mechanical ventilation (n = 32 in D28-WHO-CPS > 5 group; n = 25 in D28-WHO-CPS ≤ 5 group) or high flow oxygen (n = 13 in D28-WHO-CPS > 5 group; n = 6 in D28-WHO-CPS ≤ 5 group).

^***d***^Maximum dose of norepinephrine in µg/kg/minute.

^e^Acute kidney injury (AKI) was defined as an absolute increase of serum creatinine of ≥ 0.3 mg/dL or relatively ≥ 1.5 times of baseline creatinine.

^f^CRP and procalcitonin were available in 68 patients (n = 41 in D28-WHO-CPS > 5 group; n = 27 in D28-WHO-CPS ≤ 5 group) and 75 patients (n = 44 in D28-WHO-CPS > 5 group; n = 31 in D28-WHO-CPS ≤ 5 group), respectively.

Despite a higher frequency of acute kidney injury on ICU admission in patients with a poor 28-day outcome, the baseline severity according to the SAPS II, SOFA score, WHO-CPS, and biomarkers such as C-reactive protein and lymphopenia did not differ between the groups.

Corticosteroids, mainly hydrocortisone, were administered to most patients. No patient received antiviral drugs. Patients with a poor 28-day outcome experienced a more complicated course, with a higher incidence of ICU-acquired pneumonia and a greater need for organ support. The median length of ICU stay was 21 [13-38] days. Overall, the 28-day was 20% (for more details regarding the distribution of the study population over the classes of D28-WHO-CPS, see Table A in the ESM).

### Characteristics of BAL

Most BAL procedures (95%) were performed on intubated and mechanically ventilated patients, with a median time from ICU admission to BAL collection of 2.5 [1-6] days and a median time from intubation to BAL collection of 1 [1-4] day. The timing of BAL and specific therapies received on or before the day of BAL did not differ between groups (Table 2). Forty-one patients underwent a CT-scan in the 4 days preceding BAL (for more details regarding the investigations performed for each patient, see Table B in the ESM). Patients with a D28-WHO-CPS > 5 had a higher incidence of extensive lesions (> 50% of lung parenchyma involvement) as compared with their counterparts. The median volume of BALF recovery was 60 [50–75] mL, with no difference between groups. The BALF culture enabled the diagnosis of bacterial coinfection in 14 (18%) patients, and no additional bacterial documentation was obtained using other specimens such as blood or urine. A SARS-CoV-2 reverse transcriptase polymerase chain reaction (RT-PCR) was performed in the BALF of 43 patients. No difference was observed between groups regarding either its positivity rate or the cycle threshold (Ct) value in positive patients.

**Table 2:** Clinical and paraclinical data regarding bronchoalveolar lavage in critically ill COVID-19 patients, according to the 28-day WHO-CPS.

Patients	All patients (n = 76)	D28 WHO-CPS>5 group (n = 45)	D28 WHO-CPS≤5 group (n = 31)	P value^***a***^
**Timing of BAL**				
Time from symptoms onset to BAL (days)	13 [9–17]	13 [9–18]	12 [8–15]	0.31
**Specific therapies received on or before the day of BAL**				
Tocilizumab	8 (11)	5 (11)	3 (10)	1
Corticosteroid	52 (68)	31 (69)	21 (68)	1
Time from corticosteroid start to BAL (days)	2 [1–5]	3 [1–6]	2 [1–3]	0.1
**Chest CT-scan patterns at the time of BAL**				
Crazy paving	33/41 (80)	17/22 (77)	16/19 (84)	0.7
Consolidation	34/41 (83)	20/22 (91)	14/19 (74)	0.22
Lesions > 50% of lung parenchyma	26/41 (63)	21/22 (95)	5/19 (26)	**<0.001**
**Microbiological documentation in BALF**				
Bacteria	14 (18)	8 (18)	6 (19)	1
SARS-CoV-2 (RT-PCR)	37/43 (86)	20/24 (83)	17/19 (89)	0.68
Gene E cycle threshold (Ct)	16 [10–23]	17 [10–24]	16 [9–22]	0.55
Other respiratory virus	3 (4)	2 (4)	1 (3)	1

Data are presented as median [first through third quartiles] or number (%). Patients were grouped according to their WHO-CPS value at 28-day. A score value higher than 5 (D28-WHO-CPS > 5) defined a poor 28-day outcome, whereas a score value equal or lower than 5 (D28-WHO-CPS ≤ 5) defined a good 28-day outcome.

*BAL* bronchoalveolar lavage, *BALF* BAL fluid, *chest CT-scan* chest computed tomography scan, *ICU* intensive care unit, *RT-PCR* reverse transcriptase polymerase chain reaction, *SARS-CoV-2* severe acute respiratory syndrome coronavirus 2, *SOFA* sequential organ failure assessment.

^***a***^P values refer to differences between D28-WHO-CPS > 5 and D28-WHO-CPS ≤ 5 groups. Boldface values indicate statistically significant differences between the groups.

### Cytological findings

**Figure 2:**
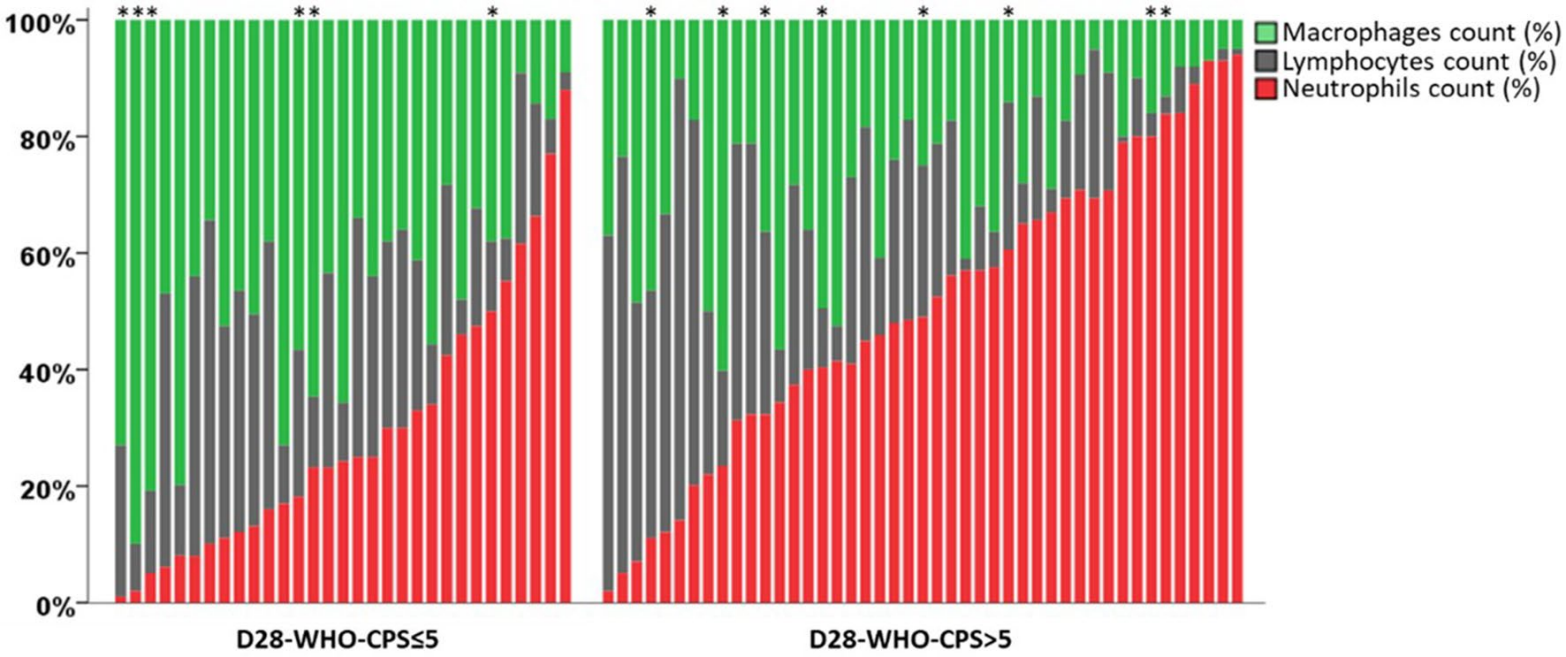
Contributions of main cellular types (macrophages, lymphocytes and neutrophils) in bronchoalveolar lavage fluid in critically ill COVID-19 patients, according to the 28-day WHO-CPS. Stacked bars represent the percentages of macrophages (green), lymphocytes (grey) and neutrophils (red) in bronchoalveolar lavage fluid (BALF). Each vertical line represents data from one single patient. Stars point out patients with a bacterial documentation in BALF. Patients were grouped according to the World Health Organization 10-point Clinical Progression Scale (WHO-CPS) at 28-day. A score value higher than 5 (D28-WHO-CPS > 5) defined a poor 28-day outcome, whereas a score value equal or lower than 5 (D28-WHO-CPS ≤ 5) defined a good 28-day outcome.

The total BALF cell count of did not differ between groups, whereas marked differences were observed in the differential cell counts (Table 3). Patients with a poor 28-day outcome displayed a neutrophil-predominant phenotype, while the others exhibited a macrophage-predominant phenotype. No obvious link was observed between the neutrophil count and the bacterial documentation in BALF (Fig. 2; see also Figure A in the ESM). The BALF and blood lymphocyte count, and the phenotyping did not differ between groups. Eventually, patients with a poor 28-day outcome displayed a much higher BALF neutrophil/lymphocyte ratio, as compared with their counterparts.

**Table 3:** Bronchoalveolar lavage fluid and blood cytological analyses in critically ill COVID-19 patients, according to the 28-day WHO-CPS.

Patients	All patients (n=76)	D28 WHO-CPS>5 group (n=45)	D28 WHO-CPS≤5 group (n=31)	P Value ^***a***^
BALF total cell count (cell/µL)	215 [130–358]	190 [130–285]	280 [130–470]	0.13
**BALF differential cell counts**				
Macrophages (%)	34 [17–46]	21 [13–36]	44 [34–56]	**<0.001**
Neutrophils (%)	41 [19–64]	49 [32–68]	24 [11–46]	**0.001**
Lymphocytes (%) ^***b***^	25 [9–34]	21 [7–34]	25 [10–36]	0.39
CD3 (%)	88 [80–94]	88 [81–93]	86 [77–96]	0.84
CD4 (%)	48 [39–52]	50 [40–63]	44 [37–51]	0.14
CD8 (%)	35 [29–41]	35 [32–39]	37 [27–48]	0.48
Ratio CD4/CD8	1.27 [0.98–1.94]	1.35 [1.08–2.25]	1.06 [0.86–1.92]	0.27
CD19 (%)	0 [0–1]	0 [0–1]	1 [0–1]	0.51
NK (%)	4 [2–8]	4 [2–8]	6 [2–9]	0.88
BALF neutrophil/lymphocyte ratio	1.80 [0.66–5.22]	2.72 [1.06–9.40]	0.88 [0.35–2.40]	**0.01**
Blood lymphocyte count (cell/µL) ^***b***^	629 [338–1097]	635 [265–1029]	629 [350–1281]	0.79
CD3 (%)	62 [53–73]	60 [52–73]	64 [54–74]	0.33
CD4 (%)	39 [28–48]	38 [28–49]	39 [28–48]	0.83
CD8 (%)	21 [15–26]	19 [14–24]	25 [16–28]	0.11
CD19 (%)	18 [10–27]	19 [8–28]	18 [11–26]	0.93
NK (%)	13 [9–21]	13 [10–18]	12 [8–23]	0.66

Data are presented as median [first through third quartiles] or number (%). Patients were grouped according to their WHO-CPS value at 28-day. A score value higher than 5 (D28-WHO-CPS > 5) defined a poor 28-day outcome, whereas a score value equal or lower than 5 (D28-WHO-CPS ≤ 5) defined a good 28-day outcome.

*BALF* bronchoalveolar lavage fluid, *ICU* intensive care unit.

^***a***^P values refer to differences between D28-WHO-CPS > 5 and D28-WHO-CPS ≤ 5 groups. Boldface values indicate statistically significant differences between the groups.

^***b***^Blood and BALF lymphocyte phenotyping (CD3, CD4, CD8, CD19, NK) were available in 46 patients (n = 26 in D28-WHO-CPS > 5 group; n = 20 in D28-WHO-CPS ≤ 5 group) and 24 patients (12 in each group), respectively.

### Cytokine findings

**Figure 3:**
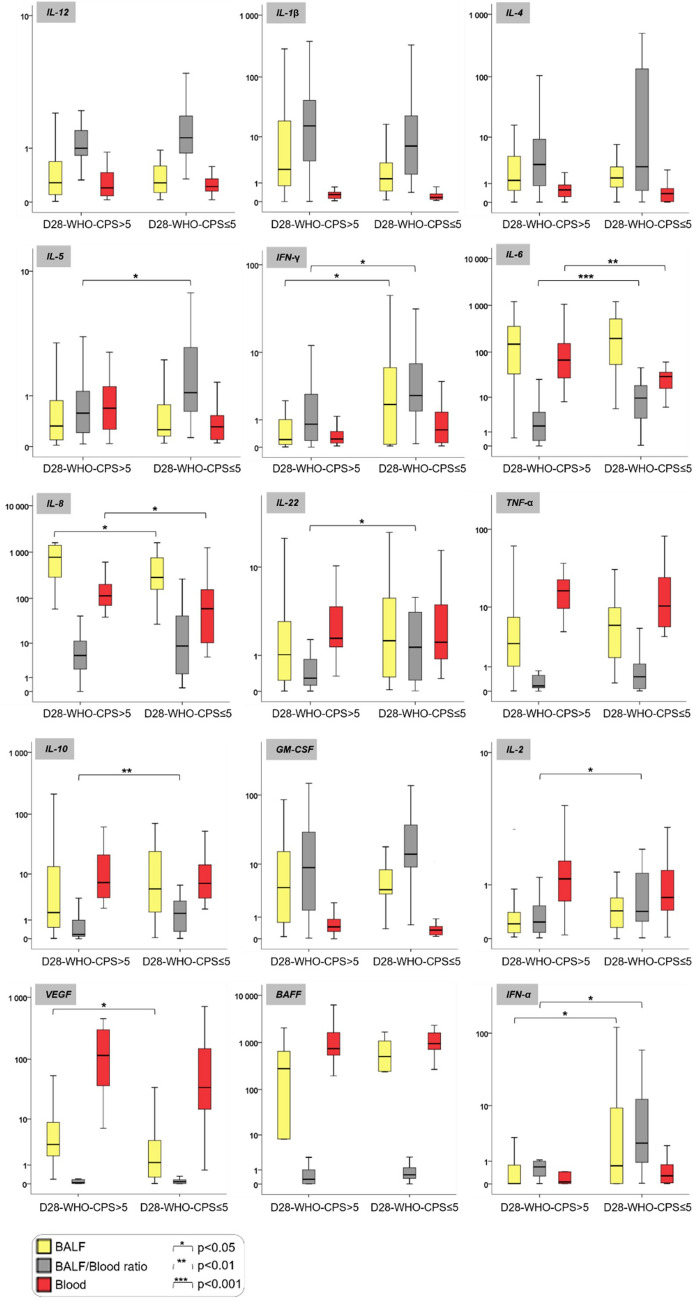
Cytokine profiling of blood and bronchoalveolar lavage fluid in critically ill COVID-19 patients, according to the 28-day WHO-CPS. Data are presented as box and whiskers plots (N = 26–32 per group). Patients were grouped according to the World Health Organization 10-point Clinical Progression Scale (WHO-CPS) at 28-day. A score value higher than 5 (D28-WHO-CPS > 5) defined a poor 28-day outcome, whereas a score value equal or lower than 5 (D28-WHO-CPS ≤ 5) defined a good 28-day outcome. Groups were compared using the non-parametric Mann–Whitney test for bronchoalveolar lavage fluid (BALF) concentrations, BALF/blood ratio of concentrations and blood concentrations of each cytokine. All the concentrations are expressed in pg/mL.

Fifteen cytokines were quantified in the BALF (n = 58) and blood (n = 52) (same-day samples) (Fig. 3). In line with neutrophil-predominant phenotypes, we observed higher BALF and blood concentrations of IL-8 (p < 0.05) in patients with D28-WHO-CPS > 5, as compared with their counterparts. We then calculated the BALF/blood ratio for the concentration for each cytokine (Figure B and C, in the ESM). Compared with patients having a good 28-day outcome, we observed in those with a poor 28-day outcome a decompartmentalized acute phase response, with a higher blood concentration of IL-6 (67.2 [22.4–156] *vs.* 29.3 [14.7–38.7]; p = 0.006) but lower BALF/blood ratios for IL-6 (1.68 [0.30–4.41] *vs.* 9.53 [2.56–19.1]; p = 0.001), IL-10 (0.17 [0.07–1.00] *vs.* 1.57 [0.26–3.10]; p = 0.003], and IFN-γ (0.79 [0.17–2.86] vs. 2.69 [1.38–7.66); p = 0.022)]. Additionally, we observed in patients with a poor 28-day outcome a lesser local type I IFN response, with a lower BALF concentration and BALF/blood ratio of concentration (p < 0.05) for IFN-α.

Regarding cytokines that reflect an adaptative immune response, we observed in patients with a poor 28-day outcome several signals that suggested a decompartmentalized adaptative response, with lower BALF/blood ratios for IL-2, IL-5, and IL-22 (p < 0.05), and a trend toward a lower BALF/blood ratio for BAFF. Finally, we quantified the growth factor VEGF in both BALF and blood, and we observed a higher BALF concentration in patients with a poor 28-day outcome compared with their counterparts (9.32 [3.29–22.8) *vs.* 4.03 [1.20–18.9]; p = 0.024).

### Multivariate analysis and eicosanoid findings

**Figure 4:**
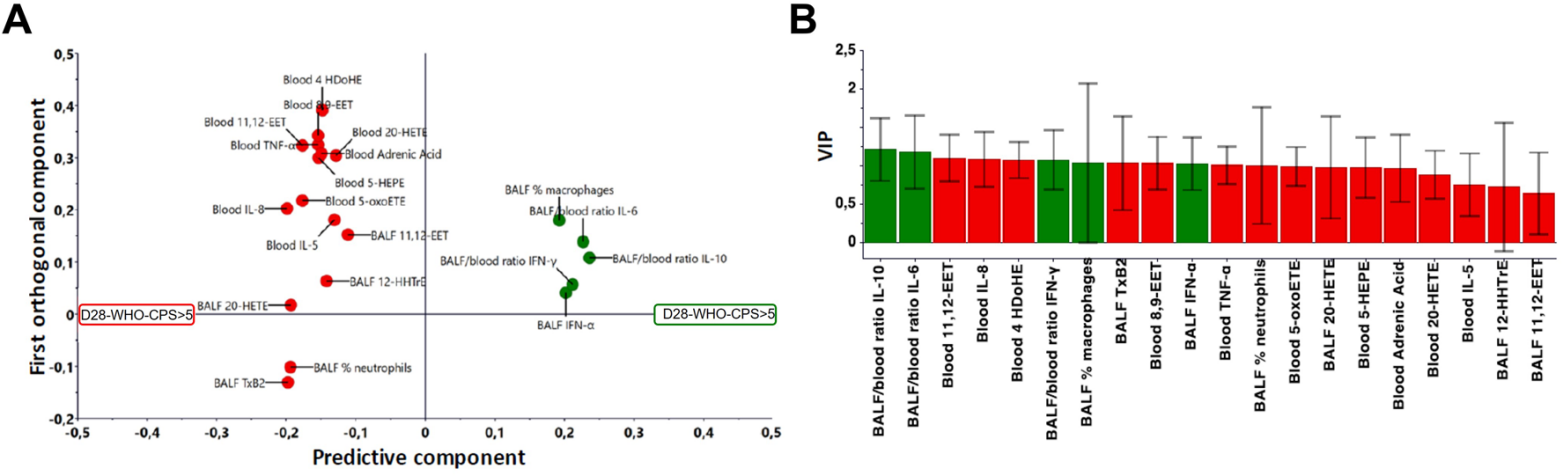
Supervised orthogonal partial least squares discriminant analysis (OPLS-DA) obtained from critically ill COVID-19 patients, according to 28-day WHO-CPS. Patients were grouped according to the World Health Organization 10-point Clinical Progression Scale (WHO-CPS) at 28-day. A score value higher than 5 (D28-WHO-CPS > 5) defined a poor 28-day outcome, whereas a score value equal or lower than 5 (D28-WHO-CPS ≤ 5) defined a good 28-day outcome. (**A**) Loading plots summarize the relationship of the 20 most discriminating variables labeled according to their association to D28-WHO-CPS ≤ 5 (green dots) and D28-WHO-CPS > 5 (red dots). (**B**) VIP (Variable Influence on Projection) plots summarize the importance of the 20 most discriminating variables. Histogram is colored according to the variable associations to D28-WHO-CPS ≤ 5 (green bars) and D28-WHO-CPS > 5 (red bars). *BALF* bronchoalveolar lavage fluid.

**Figure 5:**
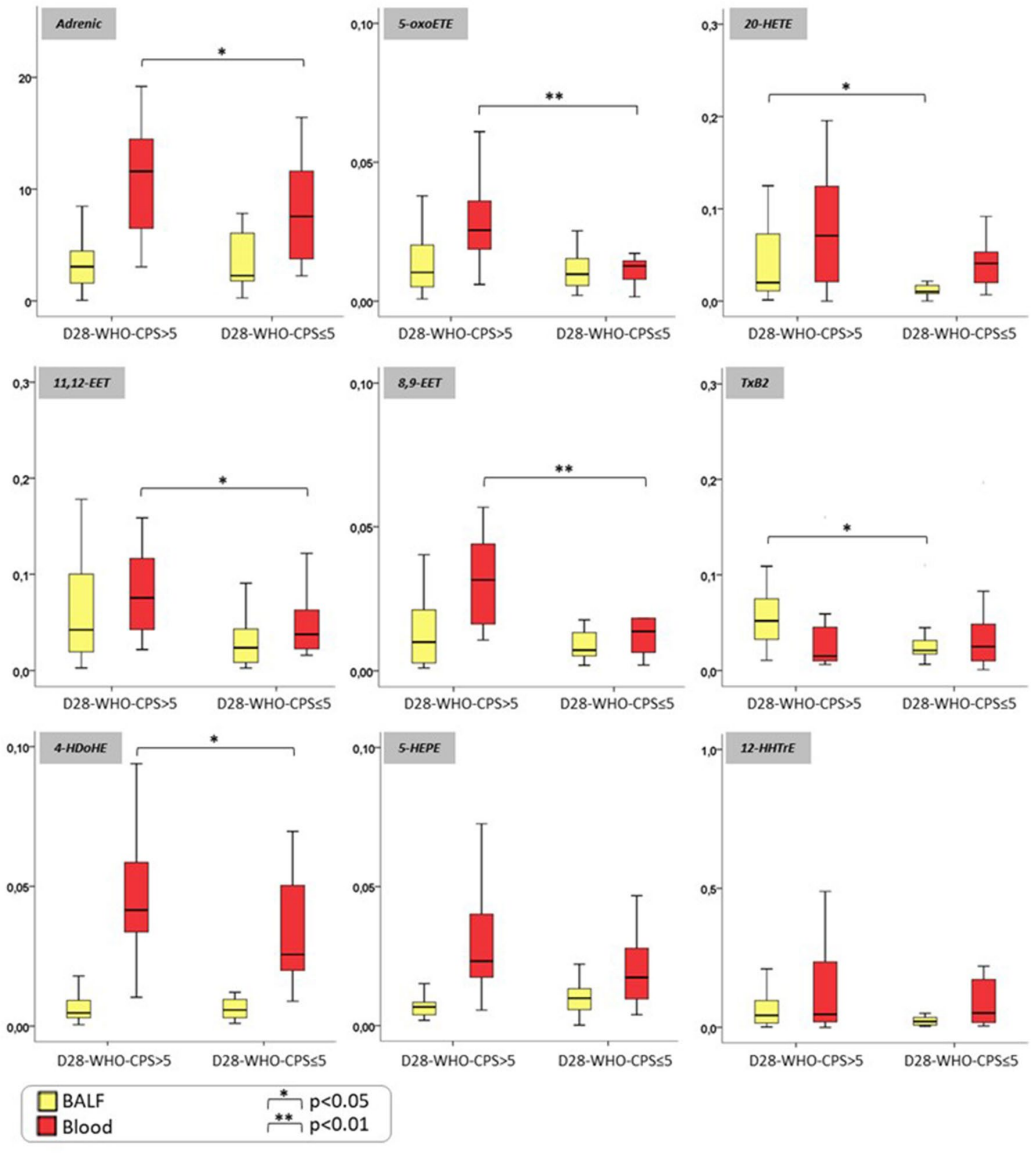
Eicosanoid profiling of blood and bronchoalveolar lavage fluid of critically ill COVID-19 patients, according to the 28-day WHO-CPS. Data are presented as box and whiskers plots (N = 16–26 per group). Patients were grouped according to the World Health Organization 10-point Clinical Progression Scale (WHO-CPS) at 28-day. A score value higher than 5 (D28-WHO-CPS > 5) defined a poor 28-day outcome, whereas a score value equal or lower than 5 (D28-WHO-CPS ≤ 5) defined a good 28-day outcome. Groups were compared using the non-parametric Mann–Whitney test for bronchoalveolar lavage fluid (BALF) concentrations and blood concentrations of each eicosanoid. All the concentrations are expressed in pg/mL.

The eicosanoid metabolomic work-up allowed to detect and quantify 107 molecular species (absolute quantification and semi-quantitative analysis of 25 and 82 molecular species, respectively) in BALF (n = 42) and blood (n = 42) (same-day samples). This inflammatory lipidome included a variety of bioactive lipid mediators that play a critical role in the regulation of inflammation. The complexity of the pathways involved led us to explore the dynamic lipidome in both the local (BALF) and systemic (blood) compartments during SARS-CoV-2 infection using liquid chromatography – mass spectrometry (LC/MS). The relative proportion of metabolites derived from arachidonic acid through cyclooxygenase (COX), lipoxygenase (LOX), and cytochrome P450 pathways, as well as metabolites derived from linoleic acid, linolenic acid, docosahexaenoic acid, and eicosapentaenoic acid were determined to assess the composition of bioactive lipids in blood and BALF. The selected panel included lipid mediators involved in pro- and anti-inflammatory processes. All the lipid metabolites, as well as the cytokines (BALF, blood and BALF/blood ratios) and cell counts were included in a multivariate analysis, which was intended to identify biological profiles associated with clinical outcomes. Multivariate analysis was restricted to the patients (n = 42) for whom all the biological data were available. The variables were screened stepwise according to their respective variable influence on projection (VIP) (Figures D, in the ESM). Among the 20 most discriminating laboratory variables (Fig. 4), 11 were eicosanoids in BALF (11,12-epoxy-5Z,8Z,14Z-eicosatrienoic acid (11,12-EET); 12S-hydroxy-5Z,8E,10E-heptadecatrienoic acid (12-HHTrE); 20-hydroxy-5Z,8Z,11Z,14Z-hydroxyeicosatetraenoic acid (20-HETE); thromboxane B2 (TxB2)) and blood (4-hydroxy-5E,7Z,10Z,13Z,16Z,19Z-docosahexaenoic acid (4-HDoHE); 8,9-epoxy-5Z,11Z,14Z-eicosatrienoic acid (8,9-EET); 11,12-EET; 20-HETE; adrenic acid; 5-hydroxy-6E,8Z,11Z,14Z,17Z-eicosapentaenoic acid (5-HEPE); 5-oxo-6E,8Z,11Z,14Z-eicosatetraenoic acid (5-oxo-ETE)), 7 were cytokines in blood (TNF-α, IL-5, IL-8), BALF (IFN-α) and the BALF/blood ratio (IL-6, IL-10, IFN-γ), and 2 were cell variables (BALF percentages of neutrophils and macrophages). The univariate comparison test focusing on eicosanoids confirmed these findings. We observed, in particular, a vascular endothelial injury eicosanoid signature in both blood and lung compartments, with higher blood concentrations of 8,9-EET and 11,12-EET (p < 0.05) and higher BALF concentrations of 20-HETE and TxB2 (p < 0.05) in patients with a poor 28–day outcome, as compared with their counterparts (Fig. 5). In addition, we observed increased blood concentrations of the neutrophil-active eicosanoids 4-HDoHE and 5-oxo-ETE (p < 0.05), as well as a tendency toward 5-HEPE (precursor of 5-oxo-6E,8Z,11Z,14Z,17Z-eicosapentaenoic acid (5-oxo-EPE)) in patients with a poor 28–day outcome, as compared with their counterparts.

## Discussion

Here, we report a series of 76 critical COVID-19 patients with immune-inflammatory marker assays on peripheral and alveolar samples, including extensive cytokine and eicosanoid analysis in 42 of them. Our findings, represented schematically in Figures E and F (in the ESM), are as follows: 1/ a neutrophil-predominant bronchoalveolar phenotype with a high neutrophil/lymphocyte ratio was associated with a poor 28-day outcome; 2/ cytokine and eicosanoid profiles were suggestive of a vascular endothelial injury and a decompartmentalized immune response, but a lesser type I IFN response in the subset of patients having a WHO-CPS value of 6 or greater at 28 days; 3/using a multi-marker approach, we identified an immune-inflammatory biomarker signature associated with the 28-day outcome.

In the cytological description of the bronchoalveolar landscape in the early stages of critical COVID-19, we identified a mixed alveolitis, with neutrophils as the main cellular type, in line with previous reports in severe SARS-CoV-2 infection^27,28^ and other virus-associated lower respiratory tract infections^29,30^. We found an association between the BALF neutrophil percentage and the clinical outcome in line with the trend observed by Pandolfi et al.^27^. In addition, we observed an elevated neutrophil/lymphocyte ratio in the BALF of patients with a poor 28-day outcome, in accordance with previous studies that identified the prognosis value of this ratio in blood in the early stages of COVID-19^31,32^.

Neutrophils are key effector cells in the acute phase response, but they may be responsible for host damage through the release of granule proteolytic enzymes, generation of reactive oxygen species, and production of extracellular traps when their local recruitment is intense and prolonged, as observed in the lungs of critically ill COVID-19 patients^33–35^. Accordingly, we observed a very high pulmonary-to-systemic gradient for IL-8, a key neutrophil chemoattractant^36^, which was almost ten times more concentrated in BALF than in blood, as previously reported^37^. In patients with a 28-day poor outcome, we observed higher BAL and blood concentrations of IL-8, in line with previous reports showing its association with COVID-19 severity^38,39^. Altogether, our findings support a marked neutrophil-predominant alveolar phenotype in patients with a poor 28-day outcome, which may participate in the dysregulated innate immune response in severe COVID-19, as reported in severe influenza infection^40^.

Qualitative anomalies of circulating neutrophils, reported as immature and/or dysfunctional, have also been described in severe COVID-19 patients^34,41–45^. In our study, the analysis of the eicosanoid metabolites brought additional insights. Two potent stimulators (5-oxo-ETE and 5-HEPE, precursor of 5-oxo-EPE) of neutrophil intrinsic functions such as migration, degranulation, and aggregation, which are produced by neutrophils themselves and act through autocrine-paracrine signaling^46^, were increased in the blood samples of patients with a poor 28-day outcome. Additionally, the blood concentration of 4-HDoHE, an eicosanoid produced from docosahexaenoic acid through the 5-lipoxygenase pathway by a wide variety of immune cells including neutrophils, showed a similar increment (p < 0.05) in patients with a poor 28-day outcome. Our findings are in line with those of Tam et al., who observed that 5-lipoxygenase-derived mediators were strongly correlated with severity in a mouse model of influenza infection^47^. Cell–cell interactions play a putative role in COVID-19-associated pulmonary vascular injury, especially interactions involving neutrophils and activated endothelial cells^48,49^. Accordingly, several biomarkers of endothelial activation and/or injury have been shown to be significantly elevated in severe COVID-19 patients; some of these biomarkers are correlated with an increased mortality^43,50–53^. In line with those findings, we observed a significantly higher BAL concentration of VEGF, a pro-angiogenic factor expressed under hypoxia, in patients with a poor 28-day outcome as compared with their counterparts. Moreover, the lipidomic analysis showed elevated BALF concentrations of the vasoactive eicosanoids TxB2 and 20-HETE in patients with a poor 28-day outcome. TxB2 is derived from TxA2, and it has been shown to play a key role in neutrophil adhesion and endothelium activation in experimental models of acute lung injury^54^. 20-HETE, one of the main cytochrome P450-dependent eicosanoids, is a hypoxia-induced mediator of oxidative stress and pulmonary arterial smooth muscle proliferation in murine pulmonary hypertension^55^. Interestingly, 20-HETE has been shown to promote endothelial dysfunction through the NF-κB signaling of vascular angiotensin-converting enzyme mRNA^56^. In our study, this eicosanoid signature of vascular injury was also evidenced in the systemic compartment, as patients with a poor 28-day outcome displayed higher blood concentrations of cytochrome P450-dependent vasoactive lipids 8,9-EET, and 11,12-EET than did their counterparts^57,58^.

COVID-19-associated pulmonary vascular injury may lead to a loss of integrity in the air-blood barrier^59^. The subsequent leak of alveolar fluid determines the loss of lung compartmentalization, as was illustrated for murine ventilator-induced lung injury decades ago^60^ and in isolated lung models^61,62^. Hyperpermeability may promote the extravasation of pro-inflammatory mediators from alveoli to the systemic compartment, ultimately leading to a marked systemic inflammatory response and subsequent multi-organ failure^63^. As an illustration, Hadjhadj et al. described a high IL-6 protein level within the blood but a low RNA level, suggesting high pulmonary production followed by massive release to the systemic compartment, especially in the most severe patients^9^. Accordingly, we described lower BAL/blood ratios for IL-6, IFN-γ, and IL-10 in patients with a poor 28-day outcome, as has been reported in patients with pneumonia-related ARDS for the main pro-inflammatory cytokines^64^.

Several limitations of our study can be underlined. First, our study was monocentric and used a number of patients that could be augmented in the future. Second, changes in therapeutic management between the first and second waves of the pandemic could be a source of variability. Third, biological work-up was not completely achieved in all patients, resulting in missing data in cytokines and eicosanoids analysis. This highlighted the difficulty to conduct translational research in a context of pandemic-driven ICU overload. Third, we chose a composite endpoint (28-day WHO-CPS), which had been previously used in cohorts of severely ill COVID-19 patients^65–67^. Indeed, considering the predictable hospital mortality, we did not choose hospital death as primary endpoint, because the relative low frequency of the event (death) would have favored the absence of difference between groups. Finally, for a better understanding of the disease, in vitro metabolomic studies on affected cells could corroborate the statistical associations between initial biological profiles and clinical outcomes.

## Conclusions

In critically ill COVID-19 patients with a poor 28-day outcome, we observed a neutrophil-predominant bronchoalveolar phenotype, a blunted type I IFN local response, and a vascular endothelial injury that resulted in a decompartmentalized immune-inflammatory response.

## Supplementary Information

Supplementary Information.

## Data Availability

De-identified individual-participant data underlying the findings described in this manuscript can be shared upon reasonable request.

## Abbreviations

4-HDoHE: 4-Hydroxy-5E,7Z,10Z,13Z,16Z,19Z-docosahexaenoic acid
5-HEPE: 5-Hydroxy-6E,8Z,11Z,14Z,17Z-eicosapentaenoic acid
5-HETE-d8: 5-Hydroxyeicosatetraenoic acid-d8
5-oxo-ETE: 5-Oxo-6E,8Z,11Z,14Z-eicosatetraenoic acid
5-oxo-EPE: 5-Oxo-6E,8Z,11Z,14Z,17Z-eicosapentaenoic acid
8,9-EET: 8,9-Epoxy-5Z,11Z,14Z-eicosatrienoic acid
11,12-EET: 11,12-Epoxy-5Z,8Z,14Z-eicosatrienoic acid
12-HETE: 12S-hydroxy-5Z,8Z,10E,14Z-eicosatetraenoic acid
12-HETrE: 12S-hydroxy-8Z,10E,14Z-eicosatrienoic acid
12-HHTrE: 12S-hydroxy-5Z,8E,10E-heptadecatrienoic acid
14(15)-EET: 14(15)-Epoxy-5Z,8Z,11Z-eicosatrienoic acid
18-HEPE: 18-Hydroxy-5Z,8Z,11Z,14Z,16E-eicosapentaenoic acid
20-HETE: 20-Hydroxy-5Z,8Z,11Z,14Z- hydroxyeicosatetraenoic acid
Adrenic: Adrenic acid
BAFF: B-cell activating factor
BAL: Bronchoalveolar lavage
BALF: Bronchoalveolar lavage fluid
CFU: Colony forming unit
CMV: Cytomegalovirus
COX: Cyclooxygenase
CT-scan: Computed-tomography scan
EDTA: Ethylenediaminetetraacetic acid
ELISA: Enzyme-linked immunosorbent assay
GM-CSF: Granulocyte–macrophage colony-stimulating factor
ICU: Intensive care unit
IFN: Interferon
IL: Interleukin
LC/MS: Liquid chromatography–mass spectrometry
LOX: Lipoxygenase
PCR: Polymerase chain reaction
RT-PCR: Reverse transcriptase polymerase chain reaction
TNF: Tumor necrosis factor
Tx: Thromboxane
VEGF: Vascular endothelial growth factor
WHO-CPS: World health organization 10-point clinical progression scale
