# Supplementary material for: Identification of bronchoalveolar and blood immune-inflammatory biomarker signature associated with poor 28-day outcome in critically ill COVID-19 patients
Source: Sci Rep. 2022 Jun 9;12:9502. doi: 10.1038/s41598-022-13179-0 (PMC9178326; doi:10.1038/s41598-022-13179-0)
Supplement: Supplementary file 1 — Supplementary Information. [file 41598_2022_13179_MOESM1_ESM.docx]

**SUPPLEMENTARY MATERIAL**

**Definitions**

Acute kidney injury (AKI) was defined as an absolute increase of serum creatinine of  ≥ 0.3 mg/dL or relatively ≥ 1.5 times of baseline creatinine, according to the KDIGO (Kidney Disease Improving Global Outcome) guidelines.

The diagnosis of ICU-acquired pneumonia was based on either i) clinical criteria (new/progressive radiological lung infiltrate together with at least two of the following: temperature >38°C or <36°C, blood leukocyte count >13 G/L or <4 G/L, or purulent respiratory tract secretion; or ii) a simplified Clinical Pulmonary Infectious Score (CPIS) ≥6 points after at least 48 hours of ICU admission in patients undergoing invasive mechanical ventilation.

**Microbiological work-up**

Urine antigen testing of *Streptococcus pneumoniae* and *Legionella pneumophila* (BinaxNOW kits, Alere, Jouy en Josas, France) and Gram staining and quantitative bacterial culture of BALF were systematically performed (threshold: 104 CFU/mL). Other microbiological investigations on BALF were on the clinician’s discretion, and may include Grocott staining, immunofluorescence and PCR (TaqMan, ThermoFischer Scientific®) for Pneumoyctis jirovecii, respiratory multiplex PCR (AllplexTM Respiratory Panel Assays, Seegene®, Seoul, South Korea), cytomegalovirus PCR (CMV R-gene kit, Argene®), and auramine-rhodamine staining and culture for mycobacteria.

**Eicosanoid metabolomic analysis**

100 µL of serum or 1 ml of BALF cell-free supernatant was supplemented with an internal standard mix consisting of 6 deuterated species (*prostaglandin D2-d4, prostaglandin E2-d4, leukotriene B4-d4, 5-hydroxyeicosatetraenoic acid-d8 (5-HETE-d8), lipoxin A4-d5, arachidonic acid-d4*) (Cayman Chemical, Interchim France).

Quantification was performed using MultiQuant 2.1 software (ABSciex). Twenty-five external standards (*prostaglandin D2, prostaglandin E2, resolvin D1, resolvin E1, 18-hydroxy-5Z,8Z,11Z,14Z,16E-eicosapentaenoic acid (18-HEPE), 6-keto prostaglandin F1α, thromboxane (Tx) B2, 11-dehydro-Tx B2, 12S-hydroxy-5Z,8E,10E-heptadecatrienoic acid (12-HHTrE), 20-hydroxy-5Z,8Z,11Z,14Z- hydroxyeicosatetraenoic acid (20-HETE), 12S-hydroxy-5Z,8Z,10E,14Z-eicosatetraenoic acid (12-HETE), 12S-hydroxy-8Z,10E,14Z-eicosatrienoic acid (12-HETrE), 14(15)-epoxy-5Z,8Z,11Z-eicosatrienoic acid (14(15)-EET), 11,12-epoxy-5Z,8Z,14Z-eicosatrienoic acid (11(12)-EET), 8,9-epoxy-5Z,11Z,14Z-eicosatrienoic acid (8(9)-EET), C16:0, C16:1, C18:0, C18:1n-9, C18:2n-6, C18:3n-3, C20:4n-6, C20:5n-3, C22:4n-6, C22:6n-3*) were assayed under the same conditions, and quantitation was achieved by the stable isotope dilution method.

External standards included two eicosanoid mixtures prepared by Cayman Chemical (Interchim France), which contained i) a collection of vasoactive eicosanoids, including the characteristic metabolites of both prostaglandin I2 and TxA2, as well as several additional hydroxyeicosatetraenoic acid and epoxyeicosatrienoic acid metabolites produced by platelets and the cytochrome P450 pathway of arachidonic acid metabolism, and ii) a mixture that comprised some species from the metabolic cascade of E-series resolvins with its principal intermediate resolvin E1, an anti-inflammatory and pro-resolving metabolite of eicosapentaenoic acid by way of an 18-HEPE intermediate.

**Table A**. Details regarding the distribution of the study population over the four classes of 28-day WHO-CPS.

| Patients | D28  WHO-CPS>5  group  (n=45) | D28  WHO-CPS≤5  group  (n=31) |
| --- | --- | --- |
| ***Ambulatory mild disease***  2 – Symptomatic; independent  3 – Symptomatic; assistance needed |  | 6 (19)  8 (26) |
| ***Hospitalized: moderate disease***  4 – Hospitalized; no oxygen therapy  5 – Hospitalized; oxygen by mask or nasal prongs |  | 13 (42)  4 (13) |
| ***Hospitalized: severe disease***  6 – Hospitalized; oxygen by NIV or high flow  7 – Intubation and mechanical ventilation, pO2/FiO2 ≥150  8 – Intubation and mechanical ventilation, pO2/FiO2 <150 or vasopressors  9 – Intubation and mechanical ventilation, pO2/FiO2 <150 and vasopressors, dialysis, or ECMO | 1 (2)  18 (40)  6 (13)  5 (11) |  |
| ***Dead***  10 – Dead | 15 (33) |  |

Abbreviations: NIV, noninvasive mechanical ventilation; ECMO, extracorporeal membrane oxygenation; WHO-CPS, World Health Organization 10-point clinical progression scale.

**Table B**. Details regarding clinical, radiological, cytological, biochemical and virological investigations performed in each of the 76 included patients (one raw per patient, in a chronological order).

| **Patient** | **Clinical data** | **Chest**  **CT-scan** | **BALF cytological analysis** | **Lymphocyte phenotyping** | | **Cytokine**  **analysis** | | **Eicosanoid**  **analysis** | | **SARS-CoV-2 RT-PCR in BALF** |
| --- | --- | --- | --- | --- | --- | --- | --- | --- | --- | --- |
|  |  |  |  | **BALF** | **Blood** | **BALF** | **Blood** | **BALF** | **Blood** |  |
| 1 | Yes | - | Y | - | - | - | - | - | - | - |
| 2 | Y | - | Y | - | - | Y | Y | Y | Y | Y |
| 3 | Y | - | Y | - | Y | Y | Y | Y | Y | Y |
| 4 | Y | - | Y | - | Y | Y | Y | Y | Y | Y |
| 5 | Y | - | Y | - | - | Y | Y | Y | Y | Y |
| 6 | Y | Y | Y | - | - | - | - | - | - | Y |
| 7 | Y | - | Y | - | - | Y | Y | Y | Y | Y |
| 8 | Y | Y | Y | - | - | Y | Y | Y | Y | Y |
| 9 | Y | - | Y | - | Y | Y | Y | Y | Y | Y |
| 10 | Y | - | Y | - | Y | Y | Y | Y | Y | Y |
| 11 | Y | - | Y | - | Y | Y | Y | Y | Y | Y |
| 12 | Y | Y | Y | - | - | Y | Y | Y | Y | Y |
| 13 | Y | - | Y | - | - | Y | Y | Y | Y | Y |
| 14 | Y | - | Y | - | Y | Y | Y | - | - | Y |
| 15 | Y | - | Y | - | - | Y | Y | Y | Y | Y |
| 16 | Y | - | Y | - | Y | Y | Y | Y | Y | Y |
| 17 | Y | Y | Y | - | - | Y | Y | Y | Y | Y |
| 18 | Y | - | Y | - | - | Y | Y | Y | Y | Y |
| 19 | Y | - | Y | - | Y | Y | Y | Y | Y | Y |
| 20 | Y | - | Y | - | Y | Y | Y | Y | Y | Y |
| 21 | Y | - | Y | - | - | Y | Y | - | - | Y |
| 22 | Y | Y | Y | - | - | Y | Y | - | - | Y |
| 23 | Y | - | Y | - | Y | Y | Y | Y | Y | Y |
| 24 | Y | Y | Y | - | - | Y | Y | Y | Y | Y |
| 25 | Y | - | Y | - | Y | Y | Y | - | - | - |
| 26 | Y | Y | Y | - | Y | Y | Y | - | - | Y |
| 27 | Y | - | Y | - | - | Y | Y | Y | Y | Y |
| 28 | Y | Y | Y | - | Y | Y | Y | Y | Y | Y |
| 29 | Y | Y | Y | - | Y | Y | Y | - | - | Y |
| 30 | Y | Y | Y | - | Y | Y | Y | - | - | Y |
| 31 | Y | - | Y | - | Y | Y | - | - | - | Y |
| 32 | Y | - | Y | - | Y | Y | Y | Y | Y | Y |
| 33 | Y | Y | Y | - | Y | Y | - | - | - | Y |
| 34 | Y | Y | Y | - | Y | Y | Y | - | - | - |
| 35 | Y | Y | Y | - | Y | Y | Y | - | - | - |
| 36 | Y | Y | Y | - | Y | - | - | - | - | - |
| 37 | Y | - | Y | - | - | Y | Y | - | - | Y |
| 38 | Y | - | Y | - | - | Y | - | - | - | Y |
| 39 | Y | Y | Y | Y | Y | - | - | - | - | Y |
| 40 | Y | Y | Y | - | Y | - | - | - | - | - |
| 41 | Y | Y | Y | Y | - | - | - | - | - | - |
| 42 | Y | Y | Y | Y | Y | - | - | - | - | - |
| 43 | Y | Y | Y | Y | Y | Y | Y | Y | Y | - |
| 44 | Y | Y | Y | Y | Y | Y | Y | Y | Y | - |
| 45 | Y | Y | Y | Y | Y | Y | Y | Y | Y | Y |
| 46 | Y | - | Y | Y | Y | Y | Y | Y | Y | Y |
| 47 | Y | Y | Y | Y | Y | Y | Y | Y | Y | Y |
| 48 | Y | - | Y | Y | Y | Y | Y | Y | Y | Y |
| 49 | Y | Y | Y | Y | Y | Y | Y | Y | Y | - |
| 50 | Y | Y | Y | Y | Y | Y | - | Y | Y | - |
| 51 | Y | Y | Y | Y | Y | Y | Y | - | - | Y |
| 52 | Y | Y | Y | Y | Y | Y | Y | Y | Y | - |
| 53 | Y | Y | Y | Y | Y | Y | - | Y | Y | - |
| 54 | Y | Y | Y | Y | Y | Y | Y | Y | Y | - |
| 55 | Y | Y | Y | - | - | - | - | - | - | - |
| 56 | Y | Y | Y | - | - | - | - | - | - | Y |
| 57 | Y | Y | Y | Y | Y | Y | Y | Y | Y | - |
| 58 | Y | Y | Y | Y | Y | Y | Y | Y | Y | - |
| 59 | Y | Y | Y | - | Y | Y | Y | Y | Y | - |
| 60 | Y | - | Y | Y | - | Y | Y | - | - | - |
| 61 | Y | Y | Y | Y | Y | Y | Y | Y | Y | - |
| 62 | Y | Y | Y | Y | Y | Y | Y | Y | Y | Y |
| 63 | Y | Y | Y | - | - | - | - | - | - | - |
| 64 | Y | - | Y | - | - | - | - | - | - | - |
| 65 | Y | Y | Y | - | - | - | - | - | - | Y |
| 66 | Y | - | Y | Y | Y | Y | Y | Y | Y | - |
| 67 | Y | - | Y | - | Y | Y | Y | - | - | - |
| 68 | Y | Y | Y | Y | Y | Y | Y | Y | Y | - |
| 69 | Y | - | Y | - | - | - | - | - | - | - |
| 70 | Y | - | Y | Y | Y | Y | - | Y | Y | - |
| 71 | Y | Y | Y | - | - | - | - | - | - | - |
| 72 | Y | - | Y | Y | Y | Y | Y | Y | Y | - |
| 73 | Y | Y | Y | - | - | - | - | - | - | Y |
| 74 | Y | - | Y | - | - | - | - | - | - | - |
| 75 | Y | Y | Y | - | - | - | - | - | - | - |
| 76 | Y | - | Y | - | - | - | - | - | - | - |

*
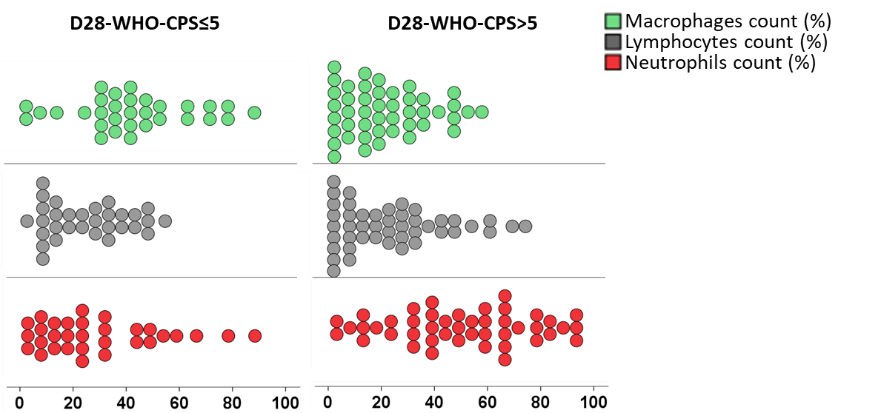
***Figure A.** Contributions of main cellular types (macrophages, lymphocytes and neutrophils) in bronchoalveolar lavage fluid in critically ill COVID-19 patients, according to the 28-day WHO-CPS.

*Figure A Legend.*

Colored circles represent the percentages of macrophages (green), lymphocytes (grey) and neutrophils (red) in BALF. Each circle represents data from one single patient. Patients were grouped according to the World Health Organization 10-point Clinical Progression Scale (WHO-CPS) at 28-day. A score value higher than 5 (D28-WHO-CPS>5) defined a poor 28-day outcome, whereas a score value equal or lower than 5 (D28-WHO-CPS≤5) defined a good 28-day outcome.

**
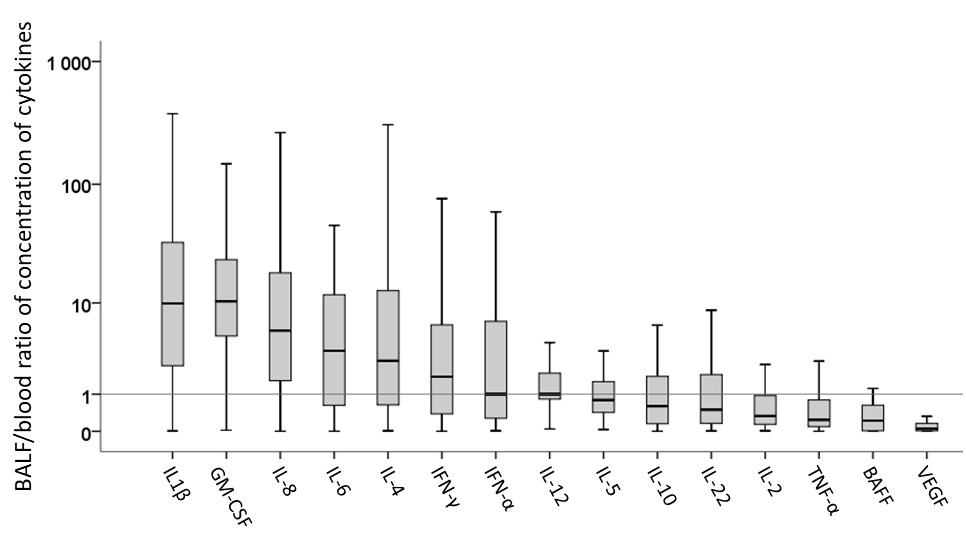
Figure B**. BALF/blood ratio of concentration of cytokines in critically ill COVID-19 patients.

*Figure B Legend.*

Data are presented as box and whiskers plots (N = 52). All the concentrations are expressed in pg/mL. Abbreviations: BALF, bronchoalveolar lavage fluid.

**Figure C.** BALF/blood ratio of concentration of cytokines in critically ill COVID-19 patients, according to the 28-day WHO-CPS.

**
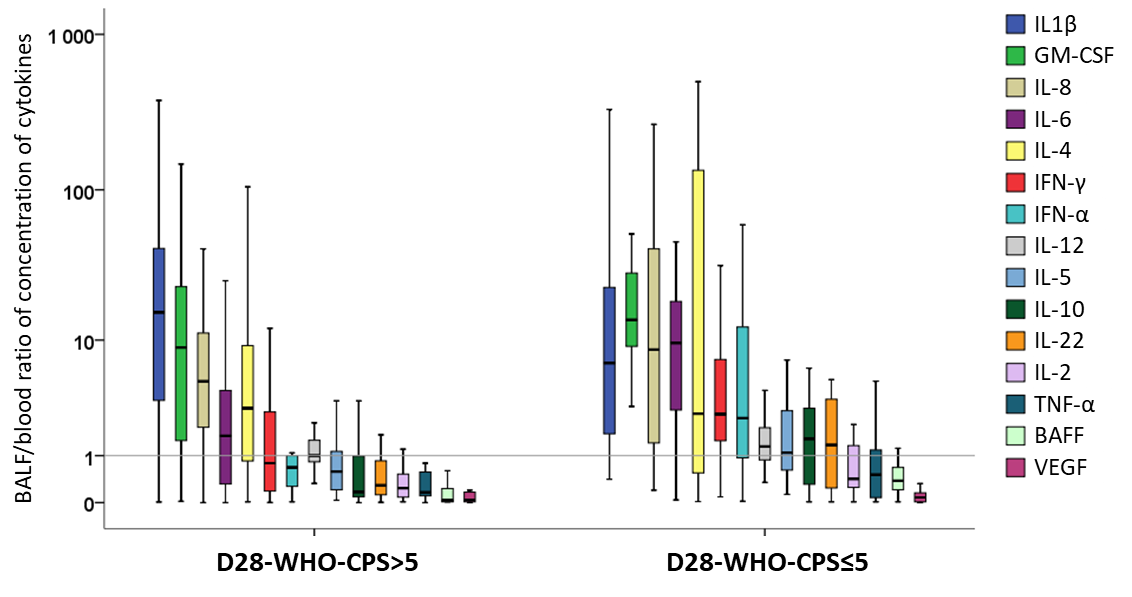
**

*Figure C Legend.*

Data are presented as box and whiskers plots (N = 52). Patients were grouped according to the World Health Organization 10-point Clinical Progression Scale (WHO-CPS) at 28-day. A score value higher than 5 (D28-WHO-CPS>5) defined a poor 28-day outcome, whereas a score value equal or lower than 5 (D28-WHO-CPS≤5) defined a good 28-day outcome. All the concentrations are expressed in pg/mL. Abbreviations: BALF, bronchoalveolar lavage fluid.


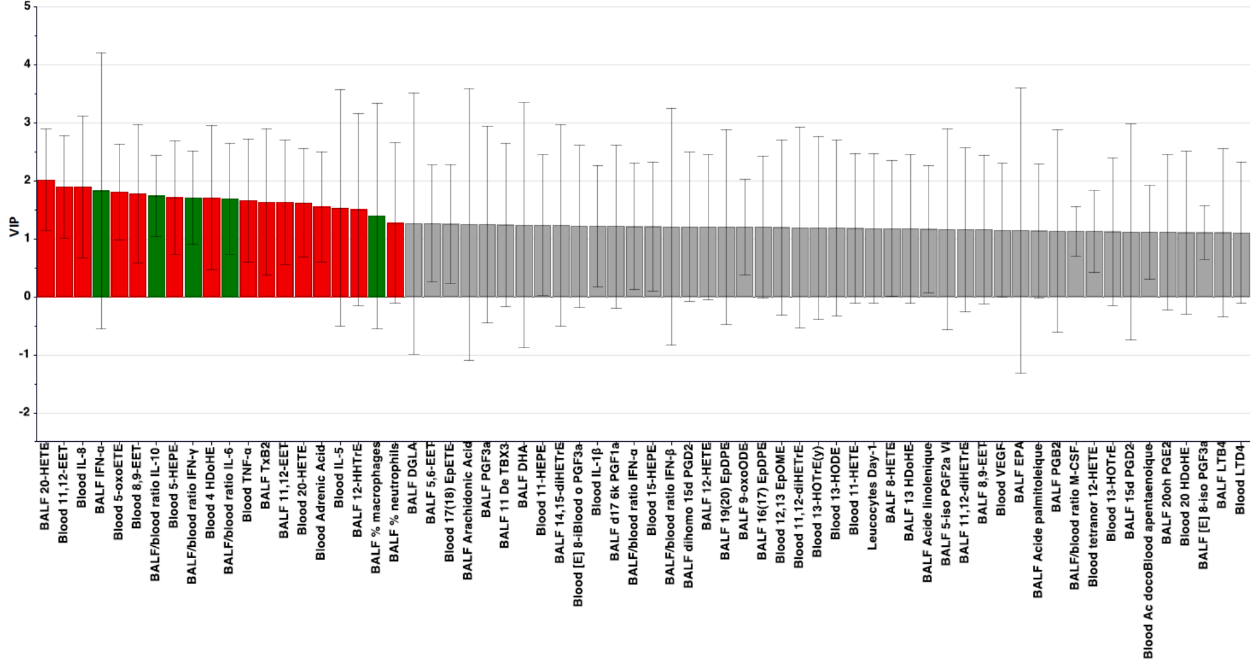

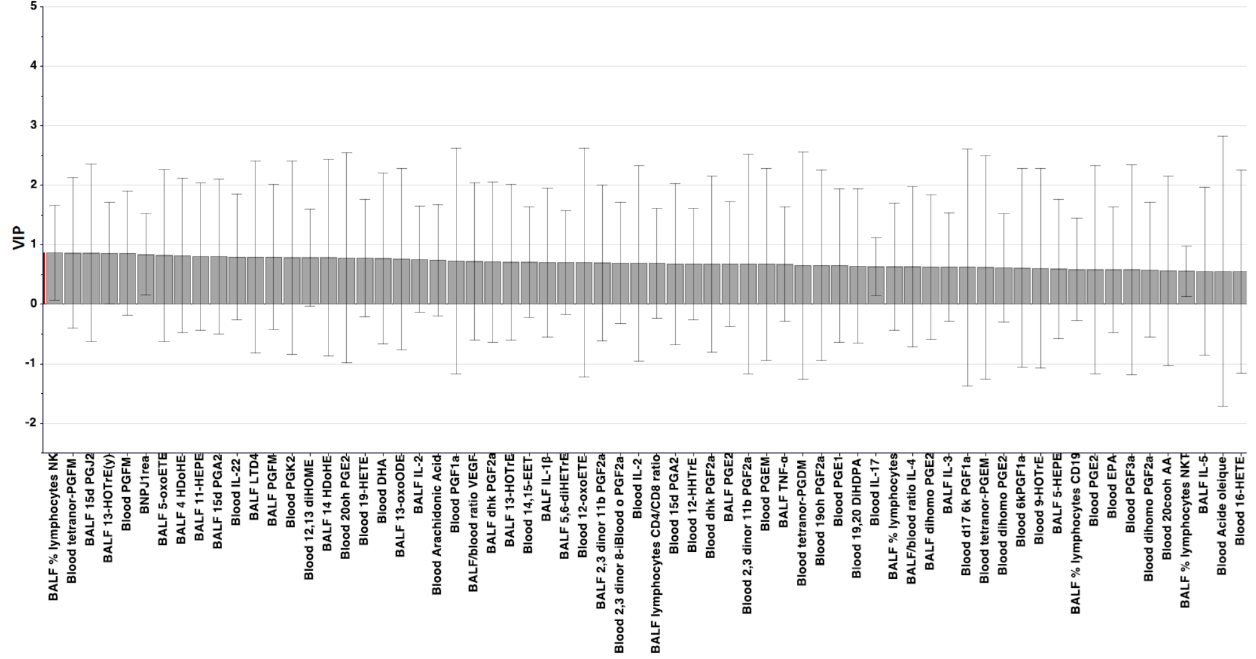

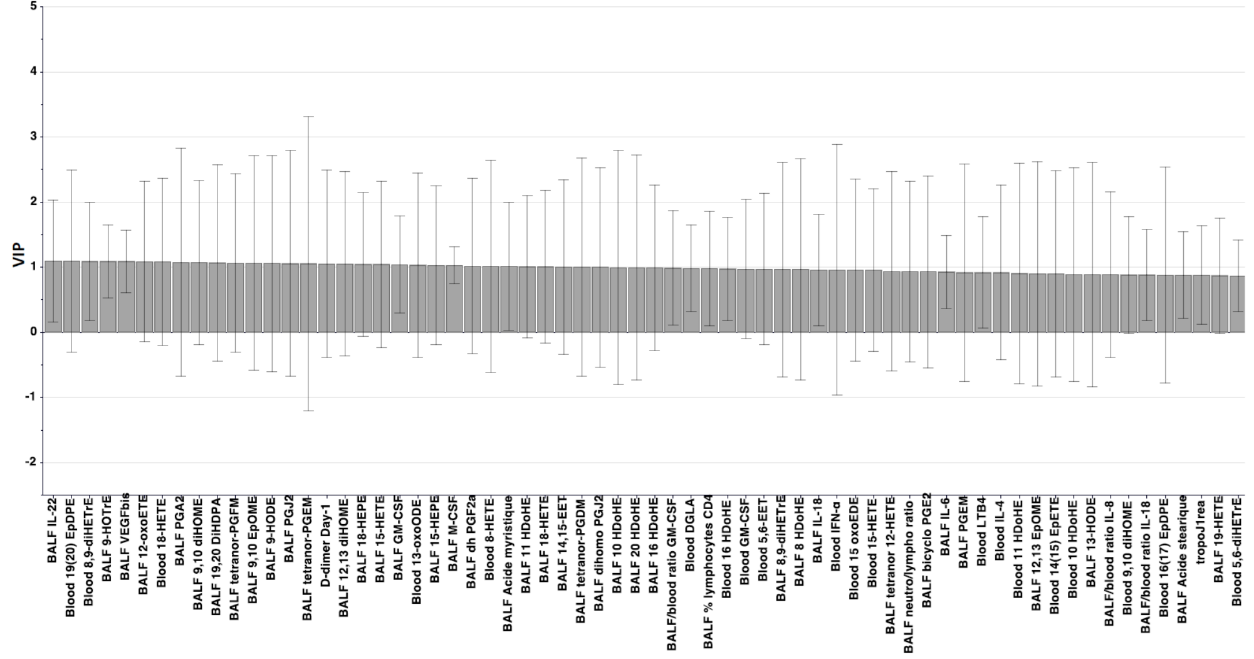
**Figure D**. Variable Influence on Projection (VIP) for all the biological variables.


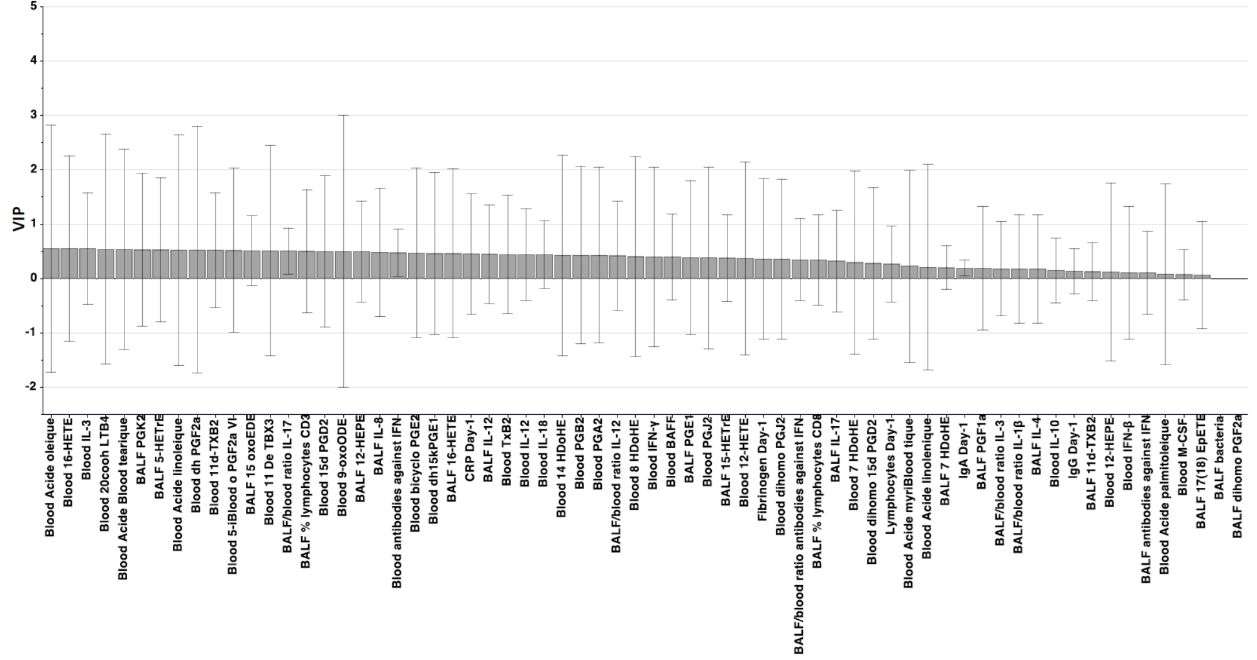
*Figure D Legend.*

The first twenty variables are labelled in green or in red according to their association to D28-WHO-CPS≤5 (green bars) and D28-WHO-CPS>5 (red bars).

*
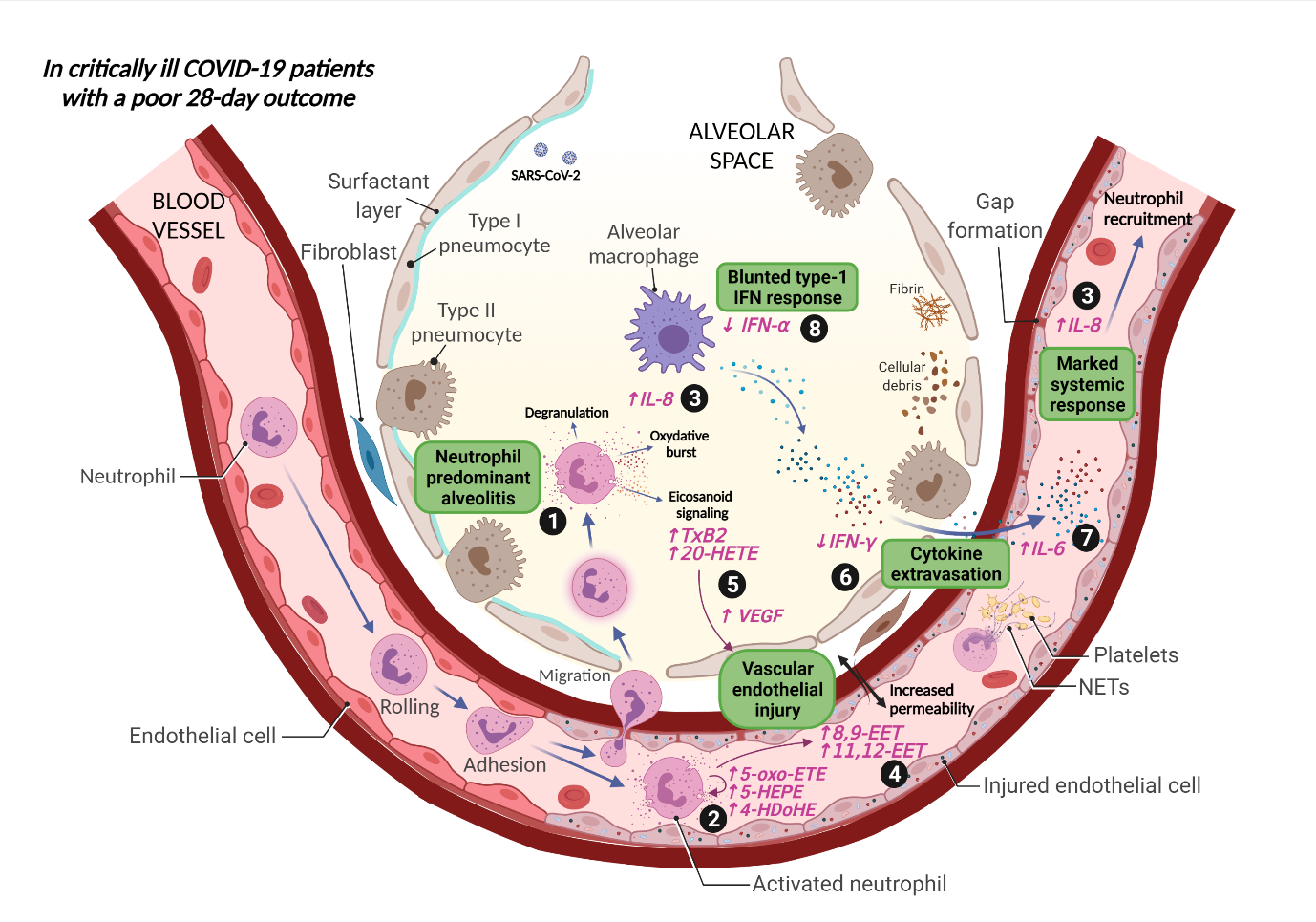
***Figure E.** Graphical synthesis.

*Figure E Legend.*

Critically ill COVID-19 patients with a poor 28-day outcome displayed a neutrophil-predominant alveolitis **(1)**, with a high neutrophil/lymphocyte ratio. Blood concentrations of the neutrophil-active eicosanoids 5-oxo-ETE, 5-HEPE (precursor of 5-oxo-EPE) and 5-HDoHE were increased **(2)**, as well as blood and BALF concentrations of the key neutrophil chemoattractant IL-8 **(3)**. This bronchoalveolar neutrophil-predominant phenotype was associated with increased blood concentrations of the vasoactive eicosanoids 8,9-EET and 11,12-EET **(4)**, and increased BALF concentrations of the growth factor VEGF and the vasoactive eicosanoids TxB2 and 20-HETE **(5)**. The subsequent vascular endothelial injury led to a hyperpermeability of the blood-air barrier, which favored a cytokine extravasation from the alveoli to the systemic compartment. As a consequence, BALF concentration of IFN-γ was lowered **(6)**, whereas blood concentration of IL-6 was increased **(7)**, and BALF/blood ratios of concentrations of IL-6, IFN-γ, IL-10, IL-2, IL-5 and IL-22 were lowered. Therefore, the immune response was decompartmentalized, with a marked systemic response, ultimately leading to organ failures. Lastly, type I interferon local response was severely blunted, with lowered BALF concentration **(8)** and BALF/blood ratio of concentration of IFN-α.

**Figure F.** Schematic synthesis .


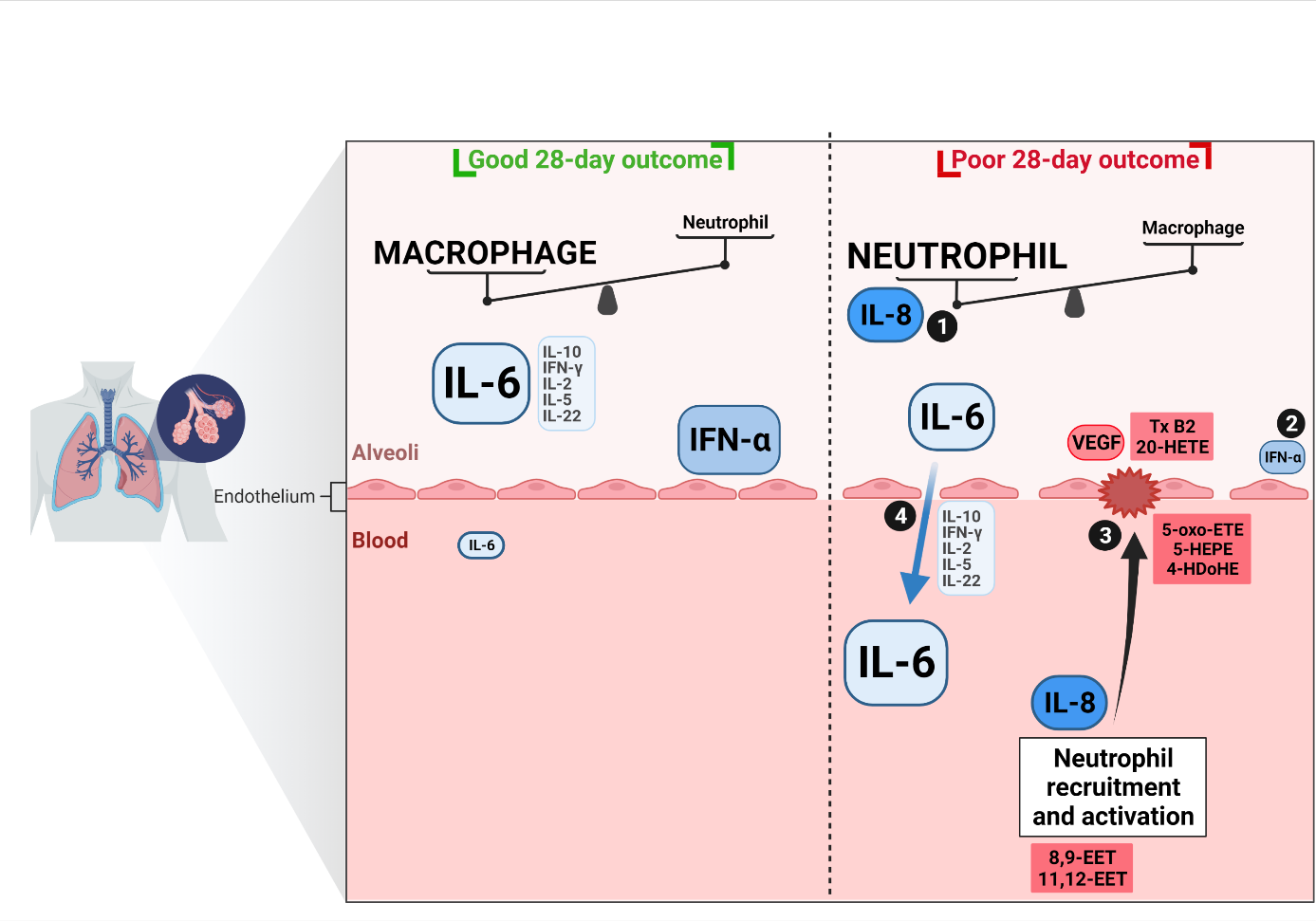


*Figure F Legend.*

In critically ill COVID-19 patients with a poor 28-day outcome, we observed a neutrophil-predominant bronchoalveolar phenotype **(1)**, a blunted type I interferon local response **(2)**, and a vascular endothelial injury **(3)**, which resulted in a decompartimentalized immune-inflammatory response **(4)** as compared to their counterparts.
